# Understanding participant perspectives around HIV-1 cure-related studies involving antiretroviral analytical treatment interruptions in the United Kingdom

**DOI:** 10.1016/j.jve.2023.100360

**Published:** 2023-12-15

**Authors:** Ming J. Lee, Piyumika Godakandaarachchi, Simon Collins, Mariusz Racz, Alice Sharp, Sarah Fidler, Julie Fox

**Affiliations:** a Department of Infectious Disease, Imperial College London, UK; b Harrison Wing, Department of HIV, Guy's and St Thomas Hospital NHS Foundation Trust, UK; c HIV I-Base, UK

**Keywords:** HIV, Treatment interruption, ATI, Antiretroviral therapy

## Abstract

**Background:**

To test efficacy, HIV cure-related trials often require a period of intensively monitored interruption of antiretroviral therapy (ART) (analytical treatment interruption or ATI). As individuals who started ART during primary HIV-1 infection (PHI) are often recruited, we have asked people already enrolled into an observational PHI study about their willingness and concerns around participating in cure-related studies involving ATIs.

**Methods:**

People who were diagnosed with PHI and started ART, attending two London HIV clinics, provided informed consent to complete a digital survey in clinic between 21/07/21 to October 31, 2023. Questions comprised sociodemographics, motivations, concerns and practical considerations influencing willingness to participate in studies involving ATIs. Hierarchical clustering of responses was performed using the ‘pheatmap’ R statistical package and ranked from most to least concerned. Responses were cross-referenced with enrolment into an ATI study which recruited from this cohort.

**Results:**

Of 352 eligible participants, 75 completed the survey. The majority were white, cisgender men who have sex with men, 34/75 (45 %) were born outside the UK. 29 (39 %) expressed interest in joining ATI studies. Participants who were interested or unsure in joining ATI studies were primarily motivated (53/65, 82 % very or moderately interested) by an altruistic desire to help scientific research. Across all participants, onward HIV transmission was the predominant concern (67/75, 89 % very or moderately concerned), and similar levels of concerns reported if the HIV-1 viral load threshold to restarting ART was increased from 500 to 50 000 copies/mL. Most participants preferred weekly (23/65, 35 %) or fortnightly (11/65, 17 %) viral load monitoring during an ATI. Before taking part in a study involving an ATI, participants stated they would prefer to discuss this with their HIV doctor (55/65, 85 %).

**Conclusion:**

In this small survey, 39 % of respondents expressed interest in joining studies involving ATIs, primarily for altruistic reasons. Participants were more interested in joining a potential ATI study if a novel intervention was included than simply an ATI alone. The main concern expressed was risk of viral transmission. To inform practical and study design considerations for future ATI studies, unrestricted access for mitigation of transmission risk should be included, and regular, frequent viral load monitoring is preferred.

## 1 Background

Whilst antiretroviral therapy (ART) blocks ongoing viral replication to levels below the limit of detection by most routine assays (less than 20 copies HIV-1 RNA/mL), it fails to cure HIV-1 infection - a consequence of a pool of latently infected cells termed the HIV-1 reservoir. This reservoir is the target of novel drugs to try to find a cure for HIV-1 which remains a high priority for people living with HIV^1^ and a realised potential among researchers^2^^,^^3^

Whilst there are many laboratory approaches to measure the HIV-1 reservoir,^4^^,^^5^ none have been demonstrated to successfully predict the most important outcome, which is time to viral rebound upon stopping ART. Hence, the only accurate way to evaluate experimental HIV cure-related interventions is to interrupt ART, a so called analytical treatment interruption (ATI).^6^^,^^7^

Stopping ART runs contrary to clinical guidance and although safe if done within a study, it does offer unique considerations particularly concerning the viral load - frequency of monitoring, onwards transmission risk and subsequent viral suppression upon ART restart. In addition, the intervention itself may have significant side effects.^8^, ^9^, ^10^, ^11^ All these factors are important to discuss at enrolment into an HIV-1 cure study. For the UK, free access to pre-exposure prophylaxis (PrEP) for all people at risk of HIV-1 has been available since 2022, enabling a more rapid and clear pathway to providing support to partners of those participating in trials.

Understanding the experiences, attitudes, and concerns around participation in HIV-1 cure trials is vital to designing studies that are acceptable to potential participants living with HIV-1, enhance recruitment and minimize anxieties for those who participate. Specific to ATI trials is to design ways and frequency of viral load measurements during an ATI which is feasible and acceptable to participants and researchers. Previous studies have surveyed the acceptability of ATI studies suggesting people living with HIV were motivated to join cure-related studies for altruistic motivations,^12^^,^^13^ but risk of onward HIV-1 transmission was a predominant concern^13^, ^14^, ^15^ in undertaking ATI studies, even when these studies were undertaken in the era of access to PrEP. However, it remains unclear how concerns around participating in ATI studies and other practical considerations may influence willingness to participate in studies involving ATIs. Due to the presence of a smaller latent HIV-1 reservoir size and diversity and higher likelihood of demonstrating post-treatment control,^16^ there is an increasing focus on people diagnosed and treated in primary HIV-1 infection (PHI) to be included in ATI studies. People diagnosed in PHI may have different areas of ethical concerns compared to those with chronic HIV-1 infection when considering recruitment into clinical trials involving ATIs, such as the urgency of recruitment, transmission risks and partner protection, and ancillary care required.^17^

By carrying out a survey in people living with HIV diagnosed during PHI, we have aimed to identify characteristics that are associated with willingness to take part in an ATI as part of a cure trial, describe patterns of concerns for participation and offer logistical solutions to enable people to take part in ATI studies.

## 2 Methods

### 2.1 Study design and population

We have conducted a cross-sectional study of adults living with HIV-1, who had received ART since being diagnosed during PHI, attending HIV clinics at Guy's & St Thomas' NHS Foundation Trust and Imperial College NHS Trust. The survey was undertaken between July 26, 2021 to October 31, 2023. Participants were already enrolled into an observational study of people diagnosed in PHI (HEATHER study).^18^

Once consent had been obtained, participants completed an online survey investigating their attitudes towards ATIs as part of future HIV-1 cure research.

### 2.2 The HEATHER and RIO studies

The HEATHER cohort was an observational study looking at the impact of ART on the viral reservoir of people with PHI.^19^ PHI was confirmed according to one of the following criteria (a) HIV positive antibody test within six months of an HIV-1 negative antibody test, (b) evidence of viral protein or nucleic acid (p24, RNA or DNA) in the absence of detectable HIV-1 antibodies or (c) a recent infection test algorithm (RITA) assay result consistent with recent infection. Eligible participants enrolled into the HEATHER prospective observational study where blood and gut samples were donated for viral reservoir and immune function analysis. Both the HEATHER study and this survey sub-study received ethical approvals from the West Midlands – South Birmingham Research Ethics Committee (REC) (REC reference 14/WM/1104).

The RIO study is a phase 2 b double-blinded randomised controlled trial of dual long-acting broadly neutralising antibodies in people diagnosed and treated during PHI and early HIV-1 infection. The study protocol has been published^20^ and participants from the HEATHER cohort were screened for eligibility to be invited to participate in the RIO study. The RIO study received ethical approval from the London – Westminster REC (REC reference 19/LO/1669).

### 2.3 Survey design

The survey comprised questions covering sociodemographic characteristics, motivations for taking part in an ATI study (with or without experimental drugs), concerns and logistical factors affecting participation. Survey questions were developed following a review of existing literature, input from investigators experienced in carrying out ATI studies, and a community representative from HIV i-Base to ensure the questions were relevant for people living with HIV.

Participants were asked 12 initial questions covering sociodemographic characteristics, logistical factors affecting participation, and willingness to take part in an ATI study. Dependent on their answer to question 12 ‘‘Would you be interested in taking part in a HIV-1 cure study which involved a treatment interruption?”, participants were offered different sets of follow-up questions. If they answered ‘Yes’ or ‘Unsure’, they continued to complete 31 questions covering concerns, attitudes towards onward HIV transmission risk prevention, concerns around viral load thresholds for re-starting ART, supportive factors on study retention and optimal viral load frequency of monitoring during treatment interruption. If they answered ‘No’ they completed a further eight questions covering their concerns and expectations of ATI studies. Survey responses were either categorical, or divided on 3, 4, or 5-point Likert scale. The full set of survey questions and responses are available in the supplementary materials. Consensus recommendations for the conduct of ATI trials suggest it may be acceptable to use a threshold of 1000 HIV-1 copies/ml for at least four weeks to gain information of early viral setpoint following study interventions, and a higher threshold of 100 000 HIV-1 copies/ml should be the upper limit for recommending immediate ART restart for safety reasons. In this survey the thresholds asked were 500 HIV-1 copies/ml for the lower limit, and 50 000 HIV-1 copies/ml for the higher limit.^21^

### 2.4 Statistical analyses

Statistical analyses were performed using R version 4.22 (22-10-31). Outcome measures were calculated for all items as univariate frequencies and proportions, compared using the Wilcoxon rank-sum test/*t*-test, fisher's exact test as appropriate to compare continuous non-parametric, parametric or categorical data. Differences in distribution were compared using the Wilcoxon rank-sum test. Demographics and responses to factors influencing participants' interest in taking part in ATI studies were initially stratified by responses to question 12.

To determine patterns of concerns around taking part in an ATI study, hierarchical clustering of responses to the following questions was performed: ‘If ART was restarted as soon as your viral load became detectable (>500 copies/mL), how concerned would you be about the following things happening?’ if they answered ‘Yes’ or ‘Unsure’ to Question 12, and ‘What are your main concerns about taking part in a treatment interruption study?’ if they answered ‘No’ to Question 12. Hierarchical clustering was performed using the ‘pheatmap’ R statistical package, with a Euclidean distance matrix and complete clustering method. Similar responses were grouped on a heatmap and associated dendrogram. The number of clusters was determined through visualisation of the dendrogram and verified by the elbow method. Clusters were ranked from most to least concerned. Responses were cross referenced with enrolment into an ongoing ATI study, the RIO trial.^20^

## 3 Results

Overall, of 352 eligible participants in the HEATHER cohort, 75 respondents completed this survey and among them 95 % (71/75) were male. Age, gender and ethnicity of survey participants were similar to the overall HEATHER cohort (Table 1). All survey participants were cisgender and 34 (45 %) born outside of the UK. The median age was 42 years (interquartile range 34–48 years). 68/71 (96 %) men reported having sex with men only, 2 (3 %) have sex with both men and women, and 1 (1 %) reported having sex with women only. 3/4 (75 %) women reported having sex with men only, and 1 (25 %) had sex with both men and women (Table 1). When asked ‘‘Would you be interested in taking part in a HIV-1 cure study which involved a treatment interruption?”, 29 (39 %) responded ‘yes’, 36 (48 %) ‘unsure’, and 10 (13 %) ‘no’. When stratified by these responses, demographics were similar across all groups (Table 1.). Among participants interested in participating in an ATI study, more people were interested in joining a ATI study with an interventional drug compared to undertaking an ATI only (37/64 (58 %) responded yes vs 15/64 (23 %), p = 0.0010).

**Table 1:** Characteristics of survey participants stratified by whether they were willing to participate in an ATI study (Q12).

			Would you be interested in taking part in a HIV cure study which involved a treatment interruption?		
	Overall HEATHER cohort (n = 352)	All survey participants (n = 75)	Yes (n = 29)	No (n = 10)	Unsure (n = 36)
Gender					
Male	338 (96 %)	71 (95 %)	26 (89 %)	10 (100 %)	35 (97 %)
Female	13 (4 %)	4 (5 %)	3 (12 %)	0 (0 %)	1 (3 %)
Transgender	1 (<0.1 %)	0 (0 %)	–	–	–
Ethnicity					
White or Caucasian	285 (81 %)	60 (80 %)	24 (83 %)	7 (70 %)	29 (81 %)
Black or African	19 (5 %)	3 (4 %)	1 (3 %)	0	2 (6 %)
Oriental	8 (2 %)	2 (3 %)	0 (0 %)	1 (10 %)	1 (3 %)
Ethnicities not listed above	40 (12 %)	10 (13 %)	4 (14 %)	2 (20 %)	4 (11 %)
Median age (IQR)	40 (34–48)	42 (34–48)	45 (39–49)	41 (39–43)	41 (34–49)
Place of birth					
Born in UK	152 (43 %)	41 (55 %)	14 (48 %)	4 (40 %)	23 (64 %)
Born outside UK	200 (57 %)	34 (45 %)	15 (52 %)	6 (60 %)	13 (36 %)
Male respondents: sexual partners					
Men only	NA	68 (91 %)	23 (89 %)	10 (100 %)	35 (100 %)
Men and women	NA	2 (3 %)	2 (8 %)	0 (0 %)	0 (0 %)
Women only	NA	1 (1 %)	1 (4 %)	0 (0 %)	0 (0 %)
Female respondents: sexual partners					
Men only	NA	3 (75 %)	2 (67 %)	0 (0 %)	1 (100 %)
Men and women	NA	1 (25 %)	1 (33 %)	0 (0 %)	0 (0 %)
Enrolled in RIO study		4 (5 %)	3 (10 %)	0 (0 %)	1 (3 %)

NA = Not available.

**Fig. 1:**
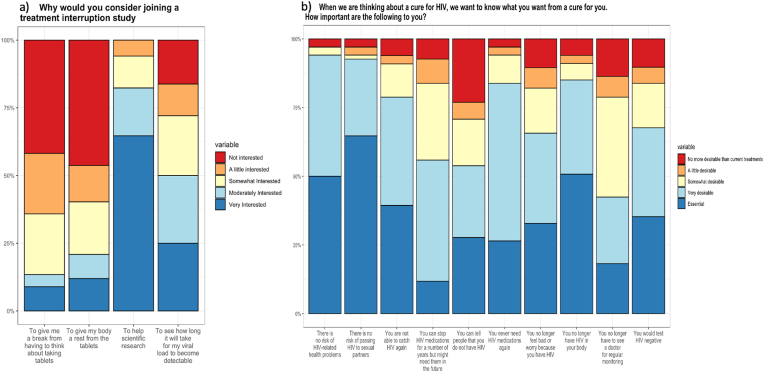
Motivations and expectations for taking part in a treatment interruption study. (n = 65, these questions were only asked for those people interested in joining clinical trials with ATIs).

Overall, the dominant motivation for considering joining an ATI study was ‘To help scientific research’, with 53 (82 %) responding either very interested or moderately interested, followed by ‘To see how long it will take for my viral load to become detectable’ (51 %) (Fig. 1a). The top three most important expectations for a cure were ‘There is no risk of passing HIV to sexual partners (92 % found it essential or very desirable), ‘There is no risk of HIV-related problems’ (94 %), and ‘You no longer have HIV in your body’ (83 %) (Fig. 1b).

**Fig. 2:**
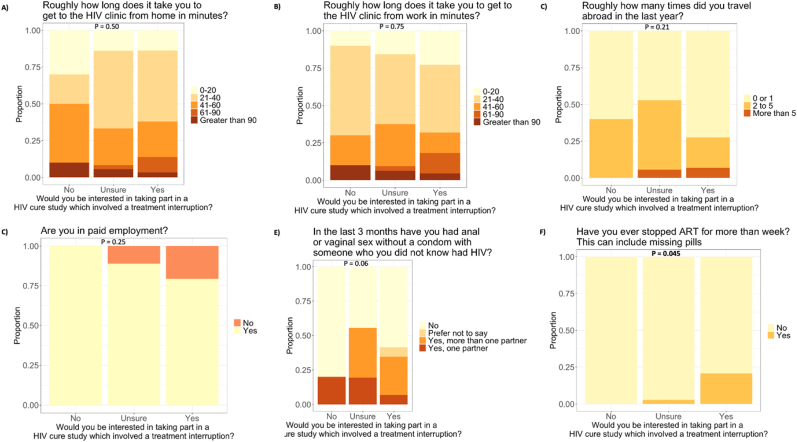
Comparing factors influencing participants' interest in taking part in studies involving treatment interruptions.

Comparing factors influencing participants’ interest in taking part in studies involving ATIs, neither commuting duration from home or work to the HIV clinic, frequency of travel abroad in the past year, being in paid employment or having had sex with someone who was not known to be living with HIV, differed across groups (Fig. 2a–e). People who were interested in ATI studies were more likely to have stopped ART previously outside of a trial setting (21 % of those who responded yes vs 3 % unsure vs 0 % who responded no to Q12, p = 0.045) (Fig. 2f). Of those who previously had stopped ART, 3/7 (43 %) had discussed their treatment interruption with their doctor.

**Fig. 3:**
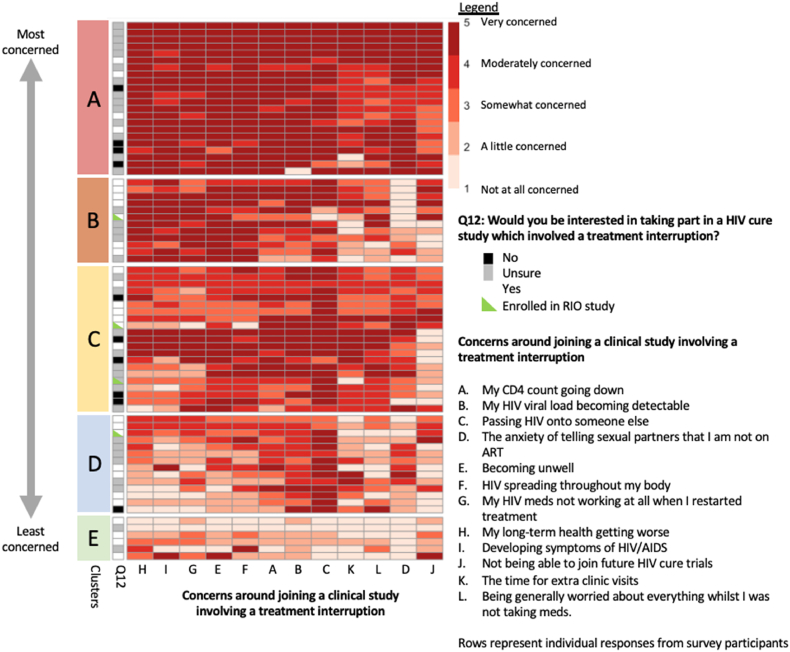
Heatmap displaying concerns of participants if they were asked to take part in a treatment interruption study.

Cluster analysis of responses to concerns around taking part in an ATI study revealed 5 groups of responses, ranked by highest to lowest levels of concerns (Clusters A-E shown in Fig. 3). These clusters were annotated with the responses to question 12, however there was no association between the demographics or responses of those who would be interested in joining clinical trials and levels of concerns by clusters (supplementary table 1). However, the four participants who did enroll into the ongoing RIO study involving an ATI had responses corresponding to the middle three clusters of concerns (Fig. 3, supplementary table 1).

**Fig. 4:**
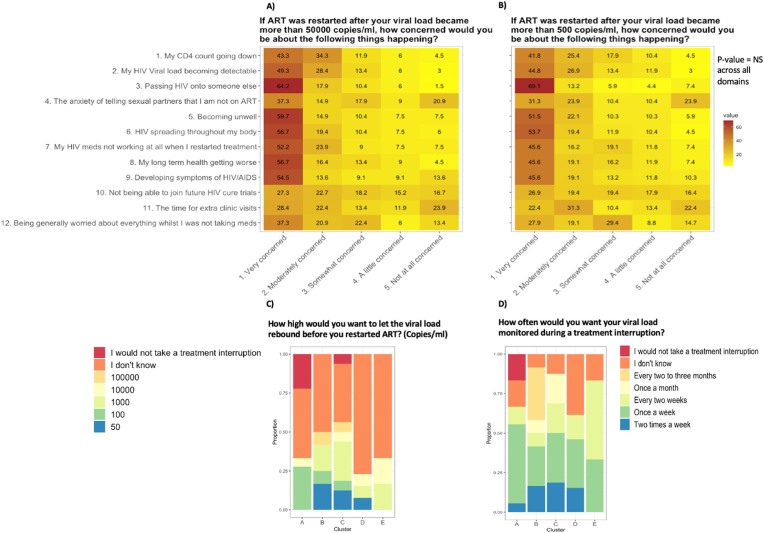
Acceptability and thresholds of undertaking a treatment interruption study (n = 65, these questions were only asked for those people interested in joining clinical trials with ATIs).

Amongst those who were interested in in joining studies involving ATIs, there was no significant shift in levels of concerns when the viral load threshold to restarting ART during an ATI was increased to 50 000 HIV-1 copies/mL (Fig. 4a) compared to 500 copies/mL (Fig. 4b). When responses to the similar question “how high would you want to let the viral load rebound before you restarted ART?” was stratified by clusters A-E, most participants reported not knowing what the appropriate threshold should be. Of the remaining responses, 1000 copies/mL was the most preferred threshold in clusters B – E (B: 33 %, C: 44 %, D: 33 %, E: 50 %), and 100 copies/mL in cluster A (83 %) with the highest levels of concerns (Fig. 4c). Most participants preferred weekly (35 %) or fortnightly (17 %) viral load monitoring during an ATI, a pattern that was consistent across all clusters (Fig. 4d).

When considering practical considerations whilst undertaking an ATI (Table 2), there was an increase in the number of people reporting being able to attend weekly clinic visits for four weeks if the clinic opening hours were increased to 8am – 6pm compared to 9am – 5pm (96 % vs 89 % Yes or maybe). 13 (45 %) of those who were interested and 13 (36 %) unsure of participating in an ATI study would be more willing to participate if they were reimbursed for the clinic visits. Before taking part in an ATI study, most participants preferred to discuss this with their HIV doctor (85 %). The most acceptable methods of monitoring viral load tests were having a blood test in clinic with results in 1 day (60/65, 92 %) or a finger prick blood test at clinic with a result in 2 h (58/64, 91 %). A blood test at clinic was preferable to a finger prick test at home with results in one day (92 % vs 73 %, p = 0.0047), and there was also a non-significantly greater preferences for finger prick test in clinic compared to having it at home with results within 2 h (91 % vs 79 %, p = 0.087) (Table 2). 54/65 (83 %) of people interested or unsure to take part in ATI studies agreed it was very important or worth considering that their partners living without HIV were using PrEP if they interrupted their treatment, and 10 (25 %) would not interrupt treatment if they had a partner living without HIV.

**Table 2:** Practical considerations for joining studies with treatment interruptions.

Would you be able to visit your clinic once a week for 4 weeks (total 4 visits) if visits were between the following times:	9am - 5pm	8am – 6pm
Yes	42 (65 %)	49 (75 %)
Maybe	16 (25 %)	14 (22 %)
No	7 (11 %)	2 (3 %)
Would you be more willing to join a study if you were paid for the clinic visits?	Q12: Yes	Q12: Unsure
More willing	13 (45 %)	13 (36 %)
Before taking part in a treatment interruption study, who would you like to talk to about it? (More than one option allowed)		
	n	%
My HIV doctor	55	85
Main partner	28	43
Friends	18	28
Family	13	20
General Practitioner or family doctor	10	15
Other regular sexual partners	5	8
Manager at work	4	6
Nobody	4	6
There may be different ways of testing your viral load. How acceptable are the following options for a treatment interruption study: (Number of acceptable or very acceptable responses)		
	n	%
Blood test at clinic with results in 5 days	30	48
Blood test at clinic with results in 1 day	60	92
Finger prick blood test at clinic with result in 2 h	58	91
Finger prick test at home with result in 1 day (not currently available)	46	73
Finger prick test at home with result in 2 h (not currently available)	50	79
I don't know	12	19

(n = 65, these questions were only asked for those people interested in joining clinical trials with ATIs).

Of those who were not interested in taking part in ATI studies, 9/10 (90 %) reported they had enough information to make an informed decision. When asked what might change their minds about taking part in an ATI study in the future, 4/10 (40 %) preferred ‘Better ways of measuring viral load at home’, 3 (30 %) preferred ‘Better cure treatments to have a treatment interruption’, and 2 (20 %) preferred ‘Better availability of PrEP for negative partners I may have during this time’.

## 4 Discussion

In our survey of people diagnosed and treated during PHI enrolled into an observational study (HEATHER), nearly 40 % of people expressed interest in participating in a future HIV-1 cure-related ATI study, and a further 48 % were undecided. Participants expressed a preference for ATI studies involving an intervention rather than undertaking an ATI-only study protocol. Willingness to participate in ATI studies was associated with prior treatment interruptions, although the reasons for doing so was not determined in this study; other practical factors, including commuting time from home or work to the clinic, or whether people were in paid employment or travelled frequently were not significantly associated with willingness to participate in ATI studies in this survey.

Participants were primarily motivated by an altruistic desire to help scientific research, which is consistent with previous studies.^12^^,^^13^ More than half were curious to see the duration during which they could remain off ART before they experienced viral rebound.

When grouping concerns around participating in ATI studies, several distinct groups could be observed, with varying levels of concerns. The predominant concern (67/75 participants reporting being at least somewhat concerned) was of passing HIV-1 onto someone else, found across all groups except group E, the cluster with the lowest levels of concerns. Other important concerns include their HI-1 viral load becoming detectable, a drop in CD4 count, HIV-1 spreading throughout their body and becoming unwell.

There was no single question predictive of interest or participation in a study involving ATIs. However, the presence of concerns across all groups was not significantly associated with a lack of willingness to participate in ATI studies in this study, suggesting that identifying and addressing concerns that participants may have, would encourage participation into ATI studies. This is supported by the fact that, of the four participants responding to the survey who enrolled in the ongoing RIO study^20^ involving an ATI, they all belonged to groups B-D with moderate levels of concerns. With data available from recent studies suggesting the individual health safety of ATIs,^22^^,^^23^ lack of impact of study interventions and ATIs on viral re-suppression rates after restarting ART,^24^ and lack of evidence for an increase in the HIV-1 reservoir from short ATIs,^25^ this data should be included in patient information documents given to potential participants to address these concerns.

In terms of practical considerations of undertaking an ATI study, participants expressed a preference for extended hours available for them to undertake their regular monitoring visits during ATI, which may allow them to fit visits and minimize the visit burden on their daily schedules. Only a third of people who reported being unsure about participating in ATI studies reported that being paid for their clinic visits would make them more willing to consider, suggesting concerns other than financial impact was more important in their decision to participate. However, most participants in this survey reported being in paid employment.

Weekly or fortnightly HIV-1 viral load monitoring was preferred by most survey participants considering an ATI, and 9 in 10 participants preferred to have their viral load monitoring done in clinic, and there was no difference in preference for phlebotomy or a finger-prick test as long as there was a rapid turnaround within a day. There did not appear to be an increased preference of having a test at home, however these responses may reflect the lack of a validated test available for home testing or home sampling. In a previous study by Lau et al.,^14^ half of the participants reported they would be more willing to undergo ATI if home-based testing was available.

Decisions around potential participation in a future ATI trial were not significantly influenced by the viral load threshold to restart ART. However, there appeared to be a greater proportion of participants who would accept remaining off ART to a higher viral load with close frequent monitoring. This is in contrast to previous reports where a third of participants in other studies would not accept any periods of sustained viraemia.^13^^,^^14^ This may reflect the difference in cohorts and settings, participants in the HEATHER cohort were treated in PHI and already engaged in clinical research having previously consented for participation in an observational study, whilst those surveyed in other studies were not enrolled into research studies. The HEATHER survey was conducted in a setting where PrEP is widely accessible without cost from sexual health clinics within the UK for people at risk of HIV acquisition. Together with data from ‘Undetectable = Untransmissible) (U=U) campaigns and studies which has unequivocally shown zero risk of HIV-1 transmission when viral loads remain undetectable <200 copies/mL,^26^, ^27^, ^28^ this may have contributed to the increased willingness to undertake ATI studies with higher thresholds for restarting ART in this study. When participants were asked about their concerns around an ATI study with a viral load threshold to restart ART at 500 HIV-1 copies/mL compared to a higher threshold of 50 000 HIV-1 copies/mL, there was no significant shift in the levels of concerns across all domains, suggesting with adequate communication of the justification of undertaking a higher threshold as well as mitigation steps to address concerns of participants, a higher threshold is likely to be as acceptable as a lower threshold for participants willing to participate in ATI studies. A higher threshold would provide a greater window for studies of immunological therapies to determine their impact on viral setpoints following rebounds, as periods of higher-level viraemia may be observed before re-suppression may be observed.^21^^,^^29^^,^^30^ Regardless of ART restart threshold, researchers should consider prevention of HIV acquisition of partners without HIV, and the ethical challenges of doing so are comprehensively covered by Gilbertson et al.^17^ Approaches to partner protection have been proposed for participants in ATI studies.^11^^,^^31^

When planning future cure-related studies, this study contributes important data to inform the following recommendations: 1) The need to address risk of onward HIV-1 transmission, including access to PrEP if not freely available, and rapid turnaround times for HIV-1 viral load monitoring results. 2). Engagement with potential participant's regular HIV clinicians and other trusted sources as 85 % of people surveyed would like to speak to their regular HIV doctor prior to enrolling in an ATI study.

There were several considerations that may limit the generalisability of this study to the wider population living with HIV-1. Firstly, the study sample size was small and only included participants from two centres in the UK, and the analysis is largely descriptive for this reason. Participants were not asked about acceptable durations of viraemia in this survey. Information such as level of highest education, income scale, socioeconomic deprivation indices were not available. Although only one fifth of the HEATHER cohort responded to this survey, and participants were mostly white and men who have sex with men, this reflects the wider cohort of people diagnosed in PHI. However their attitudes and concerns to participating in clinical trials may differ from the wider population of people living with HIV. The participants were already enrolled in an observational study of people diagnosed in PHI, which may have introduced a selection bias for people who were interested in participating in research.

In conclusion, this study reports the primary concerns of people diagnosed in PHI enrolled into an observational study considering participating in ATI studies. Patterns of concerns were complex and no one question could predict interest in participating in ATI studies. Engaging participant's regular HIV clinicians and other trusted sources is preferred by potential participants and should be considered by future trial investigators, and the results of this paper will inform practical and study design considerations for future ATI studies.

## Funding

This work was supported by the British HIV Association; and the UK Medical Research Council, United Kingdom.

## CRediT authorship contribution statement

**Ming J. Lee:** Conceptualization, Data curation, Formal analysis, Investigation, Methodology, Writing – original draft, Writing – review & editing. **Piyumika Godakandaarachchi:** Data curation, Formal analysis, Writing – original draft, Writing – review & editing. **Simon Collins:** Conceptualization, Writing – original draft, Writing – review & editing. **Mariusz Racz:** Data curation, Investigation. **Alice Sharp:** Conceptualization, Formal analysis, Project administration, Writing – original draft, Writing – review & editing, Methodology. **Sarah Fidler:** Conceptualization, Writing – original draft, Writing – review & editing. **Julie Fox:** Conceptualization, Data curation, Formal analysis, Investigation, Methodology, Writing – original draft, Writing – review & editing.

## Declaration of competing interest

The authors declare the following financial interests/personal relationships which may be considered as potential competing interests: MJL is funded by the UK 10.13039/501100000265Medical Research Council Clinical Research Training Fellowship. All other authors have no other relevant competing interests to declare.

## Data Availability

Data will be made available on request.
