# Supplementary material for: Understanding participant perspectives around HIV-1 cure-related studies involving antiretroviral analytical treatment interruptions in the United Kingdom
Source: J Virus Erad. 2023 Dec 15;9(4):100360. doi: 10.1016/j.jve.2023.100360 (PMC10770752; doi:10.1016/j.jve.2023.100360)
Supplement: Multimedia component 1 [file mmc1.docx]

**Supplementary Table 1. Characteristics of survey participants by clusters of responses to concerns around ATI studies**

|  | Cluster A  Most concerned  (n=22) | Cluster B  (n=12) | Cluster C  (n=21) | Cluster D  (n=14) | Cluster E  Least concerned  (n=6) | p-value |
| --- | --- | --- | --- | --- | --- | --- |
| Gender |  |  |  |  |  | 0.66 |
| - Male | 20 (91%) | 11 (92%) | 21 (100%) | 13 (93%) | 6 (100%) |  |
| - Female | 2 (9%) | 1 (8%) | 0 (0%) | 1 (7%) | 0 (0%) |  |
| Ethnicity |  |  |  |  |  | 0.063 |
| - White or Caucasian | 14 (64%) | 11 (92%) | 16 (76%) | 14 (100%) | 5 (83%) |  |
| - Ethnicities other than white | 8 (36%) | 1 (8%) | 5 (24%) | 0 (0%) | 1 (17%) |  |
| Median age (IQR) | 42 (35 – 50) | 45.5 (37.5 – 51) | 41 (32 – 47) | 40.5 (34 – 47) | 44 (41 – 44) | 0.54 |
| Place of birth |  |  |  |  |  | 0.74 |
| - Born in UK | 11 (50%) | 8 (67%) | 12 (57%) | 6 (43%) | 4 (67%) |  |
| - Born outside UK | 11 (50%) | 4 (33%) | 9 (43%) | 8 (57%) | 2 (33%) |  |
| Responded Yes to Q12 (Would be interested in taking part in a cure study involving an ATI) | 4 (18%) | 7 (58%) | 7 (33%) | 7 (50%) | 4 (67%) | 0.058 |
| Yes or unsure | 18 (82%) | 12 (100%) | 16 (76.2%) | 13 (93%) | 6 (100%) |  |
| Enrolled in RIO study | 0 (0%) | 1 (8%) | 2 (10%) | 1 (7%) | 0 (0%) | 0.60 |
